# Can Mammalian Reproductive Health Withstand Massive Exposure to Polystyrene Micro- and Nanoplastic Derivatives? A Systematic Review

**DOI:** 10.3390/ijms252212166

**Published:** 2024-11-13

**Authors:** Chiara Camerano Spelta Rapini, Chiara Di Berardino, Alessia Peserico, Giulia Capacchietti, Barbara Barboni

**Affiliations:** Department of Bioscience and Technology for Food, Agriculture and Environment, University of Teramo, 64100 Teramo, Italy; ccameranosr@unite.it (C.C.S.R.); cdiberardino@unite.it (C.D.B.); gcapacchietti@unite.it (G.C.); bbarboni@unite.it (B.B.)

**Keywords:** PS-MNPs, mammals, reproductive health, male fertility, female fertility, endocrine disruption, antioxidants

## Abstract

The widespread use of plastics has increased environmental pollution by micro- and nanoplastics (MNPs), especially polystyrene micro- and nanoplastics (PS-MNPs). These particles are persistent, bioaccumulative, and linked to endocrine-disrupting toxicity, posing risks to reproductive health. This review examines the effects of PS-MNPs on mammalian reproductive systems, focusing on oxidative stress, inflammation, and hormonal imbalances. A comprehensive search in the Web of Science Core Collection, following PRISMA 2020 guidelines, identified studies on the impact of PS-MNPs on mammalian fertility, including oogenesis, spermatogenesis, and folliculogenesis. An analysis of 194 publications revealed significant reproductive harm, such as reduced ovarian size, depleted follicular reserves, increased apoptosis in somatic cells, and disrupted estrous cycles in females, along with impaired sperm quality and hormonal imbalances in males. These effects were linked to endocrine disruption, oxidative stress, and inflammation, leading to cellular and molecular damage. Further research is urgently needed to understand PS-MNPs toxicity mechanisms, develop interventions, and assess long-term reproductive health impacts across generations, highlighting the need to address these challenges given the growing environmental exposure.

## 1. Introduction

The widespread use and disposal of plastics have led to an alarming increase in environmental pollution by micro- and nanoplastics (MNPs), which are small plastic particles ranging in size from micrometers to nanometers. These particles originate from the breakdown of larger plastic debris or are intentionally manufactured for use in products such as cosmetics, cleaning agents, and industrial processes. Among the most common MNPs, polystyrene micro- and nanoplastics (PS-MNPs) are of particular concern due to their persistence in the environment, potential for bioaccumulation, and adverse effects on biological systems [1,2,3]. Polystyrene (PS) is one of the most widely produced plastics, frequently used in packaging, insulation, and consumer products [4]. Its extensive use increases the likelihood of exposure for both humans and wildlife. Although PS’s aromatic structure is not unique among plastics, PS may still exhibit distinct biological interaction pathways due to its specific chemical configuration and environmental behavior compared to more aliphatic polymers like polyethylene and polypropylene [5]. Therefore, it is essential to study the specific impact of PS-MNPs on reproductive health to understand their broader environmental and biological implications. Although PS-MNPs are persistent in the environment, their slow degradation under environmental conditions can lead to the release of harmful substances, including endocrine-disrupting chemicals. These pollutants can leach from the plastic matrix, increasing their toxicological impact on sensitive biological systems, such as reproductive organs.

MNPs pose a significant concern to the environment and human health due to their widespread distribution and potential detrimental effects on various ecosystems [6]. A major concern is that MNPs can release endocrine-disrupting chemicals, such as residual toxic monomers, plastic additives, and other harmful substances like volatile organic compounds (VOCs) and polycyclic aromatic hydrocarbons (PAHs), which are all associated with significant risks to human health [7,8]. Some of these substances may mimic or block the normal functioning of the endocrine system, interfering with the regulation exerted by the hypothalamic–pituitary–gonadal axis [9,10,11]. As an example, even at low doses, plastic additives, such as phthalates (10–100 µg/kg/day) [9,10,11] and BPA (1–50 µg/kg/day) [12,13] have been shown to adversely affect endocrine health in both animals and humans.

Recent studies have shed light on the molecular mechanisms through which PS-MNPs exert their toxic effects. Due to their small size, nanoplastics (NPs) can penetrate biological barriers, including cellular membranes, the blood–brain barrier, and even the placental barrier, allowing them to reach critical organs and tissues [14,15,16]. The main pathways by which PS-MNPs induce toxicity involve oxidative stress [17,18,19,20], inflammatory responses [17,18,20,21], endocrine disruption [22], and bioaccumulation, often transporting other environmental pollutants, including heavy metals, exacerbating their toxic effects [23].

MNPs are now ubiquitous in terrestrial and marine ecosystems worldwide, contaminating soil, water, and even the air. In Europe, extensive monitoring has revealed the pervasive distribution of MNPs in both marine environments, such as the Mediterranean Sea, North Sea, and Baltic Sea, and terrestrial ecosystems, including agricultural soils. These plastics are transported through waterways, atmospheric deposition, and agricultural practices, contributing to environmental contamination across diverse habitats [24,25,26]. Terrestrial ecosystems are now recognized as significant reservoirs for MNPs due to plastic residues from landfills, industrial waste, and agricultural applications (e.g., plastic mulch). In marine environments, MNPs are readily ingested by a wide range of organisms, from plankton to fish, thereby entering the food web and eventually affecting humans. This widespread environmental exposure raises concerns about the bioaccumulation of MNPs and their long-term ecological impacts [23].

Recent studies have highlighted the reproductive system as a critical target for PS-MNP toxicity. Both male and female reproductive functions are vulnerable to the adverse effects of PS-MNP exposure, which can lead to reduced fertility and even infertility [27,28].

In females, PS-MNP exposure has been shown to reduce ovarian size, deplete follicular reserves, increase somatic cell apoptosis, and impair estrous cycles, culminating in reduced reproductive performance [17,18,19,29,30,31,32,33,34]. These disruptions may result from oxidative stress, inflammatory responses, and interference with endocrine signaling pathways, which are critical for maintaining ovarian function and follicle development. Additionally, evidence suggests that PS-MNPs can cross the placental barrier, potentially affecting fetal development and contributing to reproductive disorders across generations [17,18,19,29,30,31,32,33,34].

In males, PS-MNPs negatively affect sperm quality, disrupt spermatogenesis, and cause hormonal imbalances, ultimately impairing fertility [20,21,29,31,35]. Exposure to PS-MNPs leads to increased oxidative stress and inflammation in the testes, disrupting the delicate hormonal balance required for sperm production. This can result in decreased sperm motility, abnormal sperm morphology, and reduced sperm count, all of which contribute to compromised male fertility. Moreover, hormonal imbalances triggered by PS-MNP exposure further exacerbate reproductive dysfunction, affecting both the production of sperm and the overall reproductive health of males [20,21,29,31,35].

Despite the growing body of evidence on the harmful effects of PS-MNPs, significant research gaps remain. There is an urgent need for further investigations to elucidate the underlying mechanisms by which PS-MNPs disrupt reproductive health. Long-term studies are also essential to understand the chronic impacts of low-dose PS-MNP exposure across multiple generations. Additionally, research into antioxidant supplementation as a potential therapeutic strategy to mitigate the adverse effects of PS-MNPs holds promise but requires more thorough evaluation [20,30,32]. Addressing these research gaps will be crucial for developing comprehensive risk assessments and interventions aimed at protecting reproductive health from the unavoidable environmental MNP exposure. Given the increasing prevalence of plastics in the environment and the long-term consequences of exposure, mitigating these risks is a public health priority.

## 2. Methods

To address the research gaps identified, a comprehensive bibliographic search was conducted.

### 2.1. Bibliographic Search Methods

The scientific literature published in the peer-reviewed international indexes such as the Advanced Search of Web of Science (v.5.35) “Core collection” archive (https://apps.webofknowledge.com/WOS_AdvancedSearch, accessed on 1 January 2024) of the past 30 years was considered [36], using the following key words: “Microplastic”, “Nanoplastic”, “Polystyrene”, “Female Fertility”, “Male Fertility”, “Oocyte”, “Granulosa cells” “Diet”, “Oxidative Stress”, “Endocrine disruptors”, and “Ovarian Follicles”. “AND”, “OR”, and “NOT” were used as Boolean operators, and “TS” was used as a field tag.

Overall, the adopted bibliographic approach enabled the collection of 896 studies, which were then screened to gather original research focusing on the impact of nano- and microplastic exposure on female and male fertility.

### 2.2. Eligibility Criteria

The articles considered for this study were limited to those published in English between 1994 and July 2024, although relevant papers were primarily from the past 17 years. These studies specifically investigated the impact of exposure to microplastics (MPs) and NPs on maternal and reproductive health, including both male and female systems. Initially, articles were selected based on relevant titles and abstracts, excluding those primarily unrelated to fertility topics.

Articles focused on polystyrene were included if they assessed effects on oogenesis/spermatogenesis, folliculogenesis, embryogenesis, pregnancy, offspring effects, and lactation. Studies involving non-mammalian and aquatic organisms were excluded from this analysis.

### 2.3. Study Selection

After applying the search criteria, an initial count of 896 titles was obtained. Duplicate research studies were removed. All selected articles underwent comprehensive analysis, resulting in a finalized database.

### 2.4. Results

**Figure 1:**
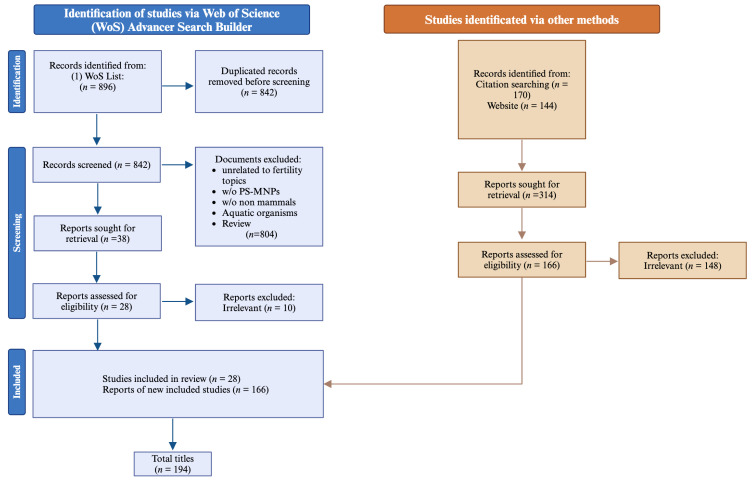
PRISMA flow diagram. The PRISMA flow diagram illustrates the method used to include articles from the literature search. The 2020 PRISMA guidelines were followed for systematic reviews. Image created with Biorender.com (accessed on 26 July 2024).

Original research articles (n = 28) were selected to investigate the impact of exposure to polystyrene micro- and nanoplastics (PS-MNPs) on specific mammalian models and to evaluate reproductive outcomes (Figure 1).

The review articles were included solely to enhance and discuss the collected data. Their reference lists were examined and analyzed, and a thorough website check was performed to identify additional studies or relevant information that could be included in this review (n = 166). Ultimately, 194 publications met the inclusion criteria (Figure 1). This systematic review has been registered in the Open Science Framework (https://osf.io/, accessed on 16 October 2024) public registry with registration number https://doi.org/10.17605/OSF.IO/AUVHX (accessed on 16 October 2024). Note that the reference related to the proper utilization of the PRISMA Statement 2020 Checklist Guidelines is excluded from the count in the systematic selection of the aforementioned articles [37].

## 3. Increased Usage of Plastic Materials, Micro- and Nanoplastic (MNPs), Sources, and Environmental Pollution

The surge in plastic production has been swift since the 1950s, with a fourfold increase in consumption over the past three decades [38,39,40]. In 2019, global plastic production reached 460 million tons, accounting for 3.4% of worldwide greenhouse gas emissions [41]. With rising global populations and incomes, plastic consumption and disposal continue to escalate. Despite concerted efforts to address the issue, policies targeting the reduction of plastic leakage into the environment are proving insufficient. The concern is heightened by projections that plastic demand will continue to rise, with production expected to reach 589 million tons by 2050 [25].

As a result, an amplified use of plastic materials has been recorded, leading to an unstoppable proliferation of MNPs within a wide range of products, raising concerns about the environmental and health impact of the widespread presence of MNPs [42].

These particles enter the atmosphere through multiple routes, including industrial activities [43], transportation [44], agricultural practices [45], and the degradation [46] of existing plastics.

Direct emissions from industrial processes and human activities are a primary source of MNPs in the air [47]. During the production and processing of plastic products, small particles are released into the atmosphere, contributing to the overall load of MNPs. Similarly, the deterioration of environmentally exposed plastic materials, such as tires and synthetic clothing [48,49], represents another important pathway of MNP release, relevant in urban areas with heavy vehicle traffic.

Once suspended in the air, MNPs can travel long distances through the wind, spreading over vast geographical areas. This widespread atmospheric transport can reach remote places, demonstrating the global extent of MNP contamination [50].

Simultaneously, MNPs can deposit on soils and water surfaces [51,52,53] contributing to ecosystem contamination.

MNP water contamination occurs through pathways like industrial and household discharges, land-to-sea transport via sewage and landfill systems, and the breakdown of larger plastic debris in aquatic environments [52].

Although MNPs in the food chain do not always cause immediate effects on aquatic life, studies show that they can be ingested by marine organisms at various trophic levels, raising concerns about bioaccumulation and long-term ecological impacts. While direct food chain transfer has not been conclusively demonstrated in all cases, evidence suggests that MNPs can move through the food web, potentially impacting species health and ecosystem dynamics over time [54,55,56].

### 3.1. Plastic Degradation Processes

The degradation of plastics, leading to the formation of micro- and nanoplastic particles (MNPs), is a critical environmental challenge. Microplastics (MPs), which range from 0.1 µm to 5000 µm in size, appear in various shapes, such as fragments, spheres, fibers, and granules [57,58]. MPs are classified as either primary or secondary based on their origin. Primary MPs are manufactured specifically for industrial or domestic purposes (e.g., as abrasives in cosmetics), whereas secondary MPs result from the breakdown of larger plastic items through physical, chemical, and biological processes [57,58,59,60]. These particles, through further degradation, eventually form nanoplastics (NPs), which are particles ranging from 1 to 1000 nm. These nanometric particles can persist in the environment for centuries, potentially affecting ecosystems [61,62,63]. Although most plastics are not biodegradable, environmental factors such as ultraviolet (UV) light, oxygen, and water contribute to their slow degradation over time [64]. These factors induce chain scission, making the material brittle, eventually fragmenting it into smaller particles, and leading to the formation of MNPs [64]. Understanding the specific pathways of plastic degradation is crucial for devising effective mitigation strategies.

Autoxidation, a key chemical degradation pathway, involves the reaction of oxygen with the polymer chain in the presence of heat, light, or other initiators, leading to the formation of peroxides and carbonyl groups. This process accelerates plastic degradation by creating weak points along the polymer chains, making them more susceptible to fragmentation [65]. While considerable research has focused on degradation mechanisms, significant gaps remain in understanding the long-term environmental fate of NPs, especially in terms of their interaction with natural processes such as sedimentation and the food chain.

Physical degradation, primarily driven by mechanical forces like wind, wave action, and abrasion, reduces the size of plastics without altering their chemical structure, facilitating the eventual formation of MNPs [66].

Thermal degradation occurs in oxygen-free environments and is a physical process that leads to fragmentation of the polymer chains, altering properties such as strength, durability, and color [67,68]. In the presence of oxygen, thermal oxidation accelerates this process and shifts from a physical to a chemical degradation mechanism through the production of organic peroxides and carbonyl groups. Despite the use of antioxidants to slow this process, oxidation remains a potent degradation pathway [50,69,70].

Chemical degradation, including hydrolytic and oxidative processes, is influenced by environmental factors such as water flow, turbulence, and other hydrodynamic conditions, pH, and temperature. Hydrolytic degradation occurs when plastics with hydrolysable bonds, such as esters, are exposed to water [71,72,73,74]. Oxidative degradation, triggered by oxygen, contributes significantly to biodegradation by forming reactive functional groups, including hydroxyl and carbonyl, on the polymer chain. However, more research is needed to understand how varying environmental conditions influence these degradation pathways, particularly in extreme environments such as the deep sea or polar regions. Photodegradation, initiated by ultraviolet radiation, plays a central role as a chemical process in breaking down plastics. UV light excites oxygen molecules, generating reactive free radicals that attack polymer chains, causing further fragmentation [58,75,76,77].

Photodegradation is especially effective in exposed environments but is less understood in regions where light penetration is limited, such as deep ocean waters or beneath dense vegetation [58,75,76,77]. Addressing these knowledge gaps could enhance our understanding of how plastic degradation rates differ across ecosystems.

Biological degradation is another key process in plastic breakdown. It involves microorganisms, such as bacteria and fungi, which produce enzymes capable of attacking plastic bonds [78,79,80,81,82]. Although promising, microbial degradation remains a relatively underexplored avenue in plastic waste management. The optimization of microbial consortia to target different types of plastics could offer a scalable solution for environmental bioremediation. However, a deeper understanding of the microbial communities involved and their interactions with environmental factors is essential to fully implementing this strategy [83]. To assess the degree of degradation, methods such as Fourier-transform infrared spectroscopy (FTIR) and differential scanning calorimetry (DSC) are commonly used. FTIR can detect changes in the chemical structure, particularly the formation of carbonyl and hydroxyl groups, which are indicative of oxidative degradation, while DSC helps analyze changes in thermal properties that reflect polymer breakdown. These techniques, along with molecular weight measurements, provide a comprehensive view of the degradation progress in plastics [84]. As plastic particles degrade, their surface area increases, expediting the release of chemical additives such as stabilizers, flame retardants, and plasticizers into the environment. These additives, while enhancing the performance and durability of plastics, have been shown to potentially harm the environment and human health through mechanisms of release and migration during the plastic life cycle, including usage, disposal, and recycling [85,86,87,88,89]. Future research should focus on understanding the relationship between the release of these additives and the fragmentation of plastics, and explore safer alternatives such as biodegradable or eco-friendly plastic additives to mitigate environmental impact [85,86].

### 3.2. MNP Composition

Plastics are macromolecular organic polymers characterized by high molecular weights and composed of a variety of elements including carbon, hydrogen, oxygen, nitrogen, sulfur, and chlorine [90]. They can also be produced from silicon atoms, known as silicone, in combination with carbon [91]. Most plastic polymers are derived from hydrocarbons found in crude oil, natural gas, and coal-fossil sources [90]. Consequently, plastics originate from petrochemical products and are manufactured by humans through an industrial process [91].

In the diverse spectrum of plastics manufactured and extensively employed, polymers such as polyethylene (PE), polypropylene (PP), polystyrene (PS), polyethylene terephthalate (PET), and polyvinyl chloride (PVC) emerge as pivotal constituents, exerting a fundamental influence across numerous industrial sectors and daily applications.

PE is widely found in bags, bottles, and containers, and represents a significant component [92,93,94]. Additionally, PP is employed in food containers, packaging, and textiles, contributing to the formation of MNPs [92,95]. PS is common in single-use products like cups, plates, and containers, and is another relevant element [18,92,96]. The presence of PET, found in plastic bottles, food containers, and textile fibers, further adds to the complexity of the issue [92,97]. Similarly, PVC is traceable in various products, including pipes, windows, coatings, and inflatable objects [92,98]. Finally, polyamides (nylon) are identifiable in synthetic fabrics, sports equipment, fishing lines, and other items [92,99].

The toxicity associated with plastics and MNPs can stem from various factors. One of these is the potential release of residual monomers from their production or the toxic additives used in plastics. For instance, bisphenol A (BPA) is a common residue found in polycarbonate products, while phthalate plasticizers used in PVC represent another concern [13,100]. Additionally, during the partial degradation of plastic, toxic intermediates can be produced. For example, the combustion of PS can generate styrenes, substances potentially harmful to human health [101]. Besides the release of toxic substances, hydrophobic plastic debris present in seawater can be absorbed and concentrated in MP fragments [102]. Although this plastic debris can act as “sponges” to remove dissolved pollutants from the water, they can also transport and release toxic substances into the surrounding environment, affecting terrestrial, aquatic, and marine ecosystems [103]. These toxic compounds, such as polycyclic aromatic hydrocarbons (PAHs), polychlorinated biphenyls (PCBs), and polybrominated diphenyl ethers (PBDEs), can thus become bioavailable to organisms, causing adverse effects on biodiversity and the health of global ecosystems [104,105,106,107].

### 3.3. Reproductive Health: Interference on Hypothalamic–Pituitary–Gonad (HPG) Axis

Endocrine disruptors, including bisphenols and phthalates, have become significant contributors to reproductive dysfunctions. While substantial research has illuminated their effects on various non-reproductive organs, their impact on the reproductive system remains a growing concern. These disruptors interfere with critical reproductive processes, such as steroidogenesis and folliculogenesis, and alter the morphology and function of reproductive organs. In women, this interference can lead to conditions like endometriosis and compromised embryo implantation, whereas in men, it manifests as impaired spermatogenesis and hormonal disruptions [9,108]. Despite these findings, many questions remain unanswered, particularly concerning the long-term and transgenerational effects of exposure to these disruptors on fertility [12,109]. The HPG axis plays a crucial role in regulating reproductive function, and disruption of this axis can have profound effects on both male and female fertility. Research has begun to uncover the mechanisms by which endocrine disruptors affect this axis, yet gaps in understanding persist, particularly regarding the potential synergistic effects of multiple environmental contaminants. There is also a pressing need to investigate how these effects vary across different life stages and exposure periods. While the effects of endocrine disruptors on reproductive health are increasingly well-documented, significant gaps remain, particularly concerning the emerging issue of MNP exposure. MNPs are pervasive in the environment and, like endocrine disruptors, have the potential to accumulate in reproductive tissues and interfere with the HPG axis [12]. However, research on MNPs is still in its early stages, and much remains to be explored regarding their interaction with known disruptors such as bisphenols and phthalates. Understanding the specific mechanisms by which MNPs affect reproductive organs represents a critical challenge for future research. Additionally, studies should focus on how chronic exposure to low doses of endocrine disruptors and MNPs might exacerbate reproductive health issues, especially given increasing concerns about fertility rates in both humans and animals [12,109]. Future research should also examine how these environmental pollutants contribute to reproductive aging and dysfunction over extended periods, with particular attention to transgenerational effects and the long-term health of future populations. Identifying these research gaps and addressing the need for comprehensive studies can help scientists better assess the full scope of environmental contaminants’ impact on reproductive health, paving the way for strategies to mitigate their harmful effects. This understanding is crucial not only for advancing reproductive medicine but also for broader public health initiatives aimed at preserving fertility and well-being amid rising environmental pressures.

#### 3.3.1. Endocrine Effects in Males: HP–Testis (HPT) Axis

According to research conducted in vivo, disruption of the HPG axis by exposure to phthalate esters and BPA may be harmful to sperm quality and function because the balance of gonadotropins and sex hormones (estradiol and testosterone) is essential for the initiation and progression of spermatogenesis.

Data from mice suggest aberrant spermatogenesis, disruption of the blood–testis barrier (BTB), low-quality semen, and bisphenol-induced DNA damage in sperm cells [110]. The likelihood that BPA may be linked to several health hazards, including infertility, is confirmed by the upregulation of proteins linked to cancer, oxidative stress, and apoptosis in germ cell cultures treated with BPA [111].

Phthalate exposure has been linked to altered sperm physiology, anti-androgenic effects, and oxidative stress in the testes in male rats and mice [10,112].

Other studies show that testicular function can be compromised in utero. In fact, mice exposed to a mixture of phthalates during pregnancy had markedly decreased serum testosterone levels, along with decreased mRNA expression of testicular steroidogenic genes (*StAR*, *Cyp11*, and *Cyp17*) and compromised spermatogenesis [113,114].

#### 3.3.2. Endocrine Effects in Females: HP–Ovary (HPO) Axis

The detrimental effects resulting from exposure to phthalates and BPA have also been observed at the level of the hypothalamic–pituitary–ovarian (HPO) axis.

BPA can mimic the action of estrogen by competitively binding with estrogen receptors (ERs), thereby increasing estrogen function. This mechanism of action leads to a change in the expression of ERs and, consequently, their target genes, resulting in dysfunction of the female reproductive system [115,116].

In several studies conducted in vivo on mice, exposure to BPA in utero has been demonstrated to disrupt ovarian folliculogenesis [117]. Specifically, it has been shown to alter the number of ovarian follicles, notably decreasing the count of preantral follicles [118] and increasing the number of atretic follicles [119]. Not only during in utero exposure but also following neonatal or adult exposure to BPA, whether using a very low dose or a high dose in the order of milligrams, irregular estrous cycles and folliculogenesis occur, along with the disruption of the preovulatory LH surge [120].

The research conducted by Mahalingam et al. demonstrates that the persistence of BPA across generations poses a significant concern for health. Their study reveals that prenatal exposure to BPA has multigenerational effects, impacting both ovarian preantral follicle availability and functionality. Indeed, deregulation of steroidogenic markers was observed. More in detail, alterations in the expression levels of key enzymes and proteins involved in the steroidogenic pathway were detected across the first two generations at various time points (from 0 up to 12 months per generation). These results underscore the potential long-term consequences of BPA exposure on future populations’ health [118].

In vitro studies have been carried out to comprehensively investigate the mechanisms underlying the various in vivo impairments affecting the reproductive system observed.

The effects of BPA have been investigated using 2D cell adhesive culture systems incorporating reproductive cells, as well as through the use of cumulus–oocyte complexes.

BPA exposure in granulosa cells (GCs) induces detrimental effects on both human and murine granulosa cells (GCs). This is evidenced by an increase in the Bax/Bcl-2 ratio, indicative of apoptotic activation and subsequent cell death. Additionally, BPA exposure results in G2-M cell cycle arrest and promotes autophagy through the AMPK/mTOR/ULK1 pathway, leading to DNA damage and ultimately cell death [118,121,122,123].

The exposure to BPA during oocyte maturation in mouse-derived COCs leads to impaired meiotic resumption and spindle abnormalities [124,125]. Consistently, additional studies have demonstrated that exposure to different concentrations of BPA (10 µM and 30 µM) in cumulus–oocyte complexes results in a dose-dependent inhibition of cell cycle progression, with fewer oocytes reaching the metaphase II (MII) stage and an increased incidence of spindle abnormalities [124,126]. BPA was shown also to interfere with chromosomal segregation, as evidenced by reduced chromosome formation in the polar body. Moreover, exposure to 10 μg/mL of BPA increases spindle abnormalities without elevating aneuploidy [126]. These findings suggest a negative impact of BPA on oocyte maturation and proper chromosomal division, potentially affecting fertility. Brieño-Enríquez et al. observed a significant increase in degeneration in human oocytes exposed to BPA for prolonged periods, indicating a potential impact on the observed decline in fertility in recent decades [127,128].

In addition to BPA, phthalates constitute another class of widely used chemical compounds that have raised increasing concerns regarding human health. In this regard, in vitro studies have shed light on a variety of adverse effects of phthalates on the reproductive system, including impacts on oocyte maturation and hormonal regulation. In vitro studies have revealed that DEHP promotes the recruitment of primordial follicles and follicle growth via the phosphatidylinositol 3-kinase (PI3K) pathway. This effect is likely mediated by its metabolite, mono-(2ethylhexyl) phthalate (MEHP) [129]. Furthermore, exposure to DEHP leads to a reduction in estradiol levels, likely due to decreased expression of aromatase [130]. Additionally, in vitro exposure of cultured mouse follicles to a mixture of phthalate metabolites upregulates aromatase, increases expression of Star, while decreasing the levels of downstream enzymes [131]. Furthermore, a phthalate mixture affects cell cycle regulators, apoptotic factors, and several receptors and receptor-associated genes, leading to the inhibition of growth in mouse antral follicles [132].

The effects resulting from phthalates influence both the morphology and functionality of the ovary. Specifically, phthalates are known to have significant impacts on ovarian tissues and functions, representing an important topic of study in the context of reproductive health. The studies conducted on female reproductive health in mice examine the impacts of exposure to phthalate substances, with particular attention to DEHP and diisononyl phthalate (DiNP). A series of negative effects emerges following this exposure, outlining a complex picture of endocrine and reproductive interferences [133,134,135,136]. Firstly, the disruption of estrous cycling represents one of the most consistent effects, with evidence of an increased incidence of the metestrus/diestrus phase compared to estrus, indicative of compromised hormonal regularity and likely imbalances in the hypothalamic–pituitary–ovarian axis. This phenomenon is associated with reduced fertility, as evidenced by an increased frequency of pregnancy loss and a significant decrease in reproductive capacity [134,136]. Additionally, studies highlight marked alterations in the population of ovarian follicles, characterized by a reduction in total follicle number, particularly at the expense of primordial follicles, which represent the primary ovarian reserve [133,135]. This decrease in ovarian reserve may lead to a premature ovarian aging directly impacting on reproductive success and fertility lifespan. Concurrently, significant variations in hormonal levels are observed, with reduced production of inhibin B and alterations in key hormone levels such as follicle-stimulating hormone (FSH) and luteinizing hormone (LH). These hormonal changes may further compromise the regulation of ovulatory processes and follicular maturation, thus influencing oocyte quality and conception chances [133,136]. Overall, these results underscore a substantial negative impact of phthalate exposure on female reproductive health, with potential long-term implications for fertility and reproductive well-being.

Despite the extensive knowledge of endocrine disruptors such as bisphenols and phthalates, the effects of MNPs remain a complex and evolving area of research. Understanding the homing of these particles to tissues and organs represents a crucial challenge in contemporary research. While the knowledge of endocrine disruptors has advanced, unraveling the microscopic-level movement and interactions of plastic particles within biological systems is still under investigation. Exploring this aspect is essential for a comprehensive assessment of the impacts of MNPs on human/animal and environmental health. Particularly in the field of reproductive medicine, this research is pivotal for a nuanced understanding of the potential implications on reproductive health.

**Figure 2:**
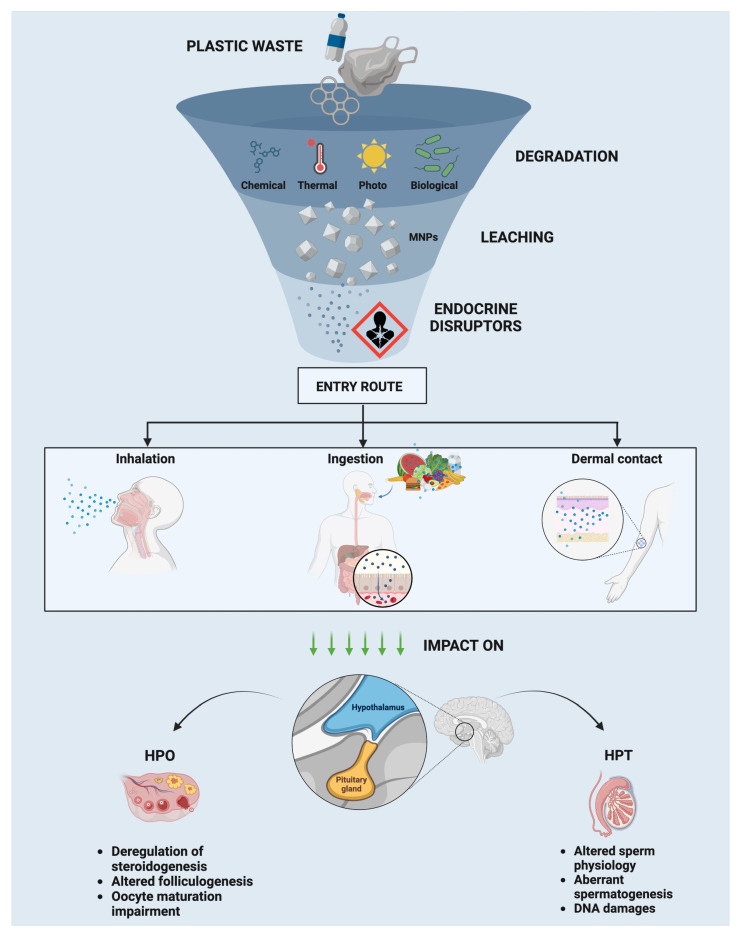
Exposure routes and endocrine-disrupting effects of MNPs on reproductive health. Overview of MNP degradation pathways, entry routes (inhalation, ingestion, dermal contact), and impacts on endocrine function, highlighting disruptions in the hypothalamic–pituitary–gonadal axis affecting both female hypothalamic–pituitary–ovary-(HPO) and male hypothalamic–pituitary–testis (HPT) reproductive systems.

An overall summary of the information described above is reported in Figure 2.

### 3.4. A Journey Along the Reproductive Effects of Polystyrene PS-MNP Exposure of Mammalian Organisms

In various studies, PS-MNPs have been tested at different concentrations to assess their potential effects on early reproductive processes in mammals. In vitro experiments typically use concentrations ranging from 1 µg/mL to 100 µg/mL [21,137,138], whereas in vivo studies have tested a broader range, including exposure levels from 0.015 mg/kg/day to 40 mg/kg/day, 0.15 µg/day to 2 mg/day, and from 1 µg/L to 1 mg/L in water-based environments [33,139,140,141] (see Table 1 for a detailed overview). Notably, most toxicological studies on microplastics have been conducted using animal models, which limits our ability to draw precise conclusions regarding human exposure. Consequently, a “minimum dose” of exposure that would cause reproductive health effects in humans has not yet been established. The range of PS-MNP exposure that negatively impacts human reproduction remains undefined, largely due to the scarcity of direct human studies on the accumulation of PS-MNPs in the body (placenta [142,143] and testis and semen [144]).

#### 3.4.1. Gestational Influence of PS-MNPs

PS-MNPs have raised significant concerns due to their potential impact on the reproductive system, as evidenced by studies demonstrating reproductive toxicity in female mice [32,33] and highlighting broader implications for human fertility, pregnancy, and child health [32,33,140,157]. By applying the One Health Concept, these concerns are relevant not only to humans but also to the animal world due to their long persistence in the environment and the fact that they can enter the body through multiple routes (including the dermis, respiratory tract, and digestive system) [158,159]. For this reason, it is crucial to limit exposure to PS-MNPs to prevent exacerbation of female reproductive syndromes and to safeguard reproductive health in women, as well as reproductive performance in livestock species and endangered species [160].

##### Impact of PS-MNPs on Pregnancy and Placental Function

Pregnancy represents a period of heightened vulnerability to environmental pollutants, as numerous studies have indicated that exposure to environmental stressors during this critical window can increase the risk of adverse health outcomes to offspring later in life [44,161,162].

Studies present in the literature have investigated the effects of exposure to PS-MNPs during pregnancy on pregnant subjects [140,147]. High concentrations of PS-MNPs have been observed to accumulate in various organs, such as maternal lung, heart, spleen, and liver [140,147]. Interestingly, in the liver, exposure to PS-NPs has been associated with induced hepatic steatosis, characterized by increased uptake and synthesis of fatty acids [140]. This observation highlights a significant metabolic impact of PS-NP exposure on liver function during pregnancy. Besides the risks associated with PS-NPs, high levels of fatty acids have been identified as an additional concern during pregnancy. Research indicates that these fatty acids can adversely affect embryo development and endometrial function, thereby compromising the implantation process and decidualization [163,164].

Reproductive organs have also been shown to be targeted by PS-MNPs. In particular, it has been shown that the accumulation of PS-MPs in the placenta during fetal development disrupts the proper functioning of the placental immune barrier [148]. Accordingly, Hu et al. demonstrated that PS-MP exposure led to a decrease in the number and diameter of uterine arterioles, accompanied by a reduction in decidual natural killer (NK) cell populations. Additionally, alterations were noted in the ratio of M1/M2 macrophage subtypes and changes in the secretion of pro- and anti-inflammatory cytokines [148].

Furthermore, it has been demonstrated that PS-MNPs can penetrate the placental barrier following acute maternal inhalation exposure. This exposure is correlated with adverse pregnancy outcomes, including decreased placental weight and an increased incidence of fetal reabsorptions [147]. The inhalation risk posed by PS-MNPs is significant, but its impact extends beyond this route. PS-MNPs can also reach the placenta via oral ingestion, as demonstrated in a study where continuous exposure to PS-MNPs throughout pregnancy via drinking water resulted in their presence within the placenta. This mode of exposure revealed evidence of placental dysfunction strongly linked to particle size, with more pronounced signs of dysfunction observed through hemodynamic indicators in the PS-NP (50 nm) group compared to other sizes of PS-MPs (5 µm) [139].

**Figure 3:**
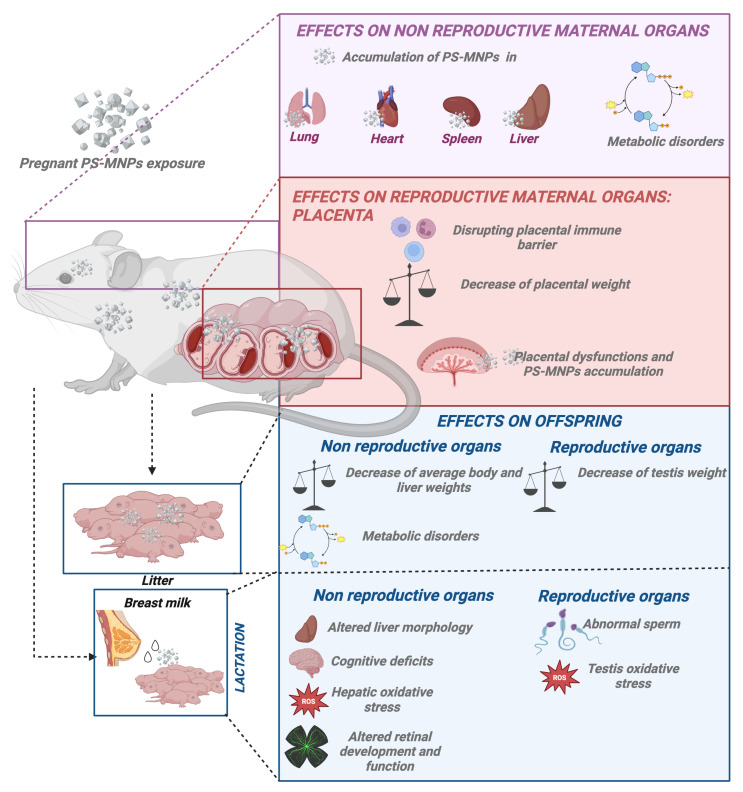
Impact of PS-MNP exposure on maternal health and offspring: Effects observed during pregnancy on placental health, offspring development, and lactation. Image created with Biorender.com (accessed on 26 July 2024).

Interestingly, a significant study assessing the effects of in vitro placental exposure provided insights into the impact of PS-MNP size on exposure toxicity. Dusza et al. confirmed that the uptake of PS-MNPs is size-dependent using non-syncytialized (undifferentiated) and syncytialized (differentiated) human placental cells. Specifically, the study observed plasma membrane damage in non-syncytialized cells exposed to 50 nm PS-NPs at high concentrations (100 µg/mL), suggesting size-specific effects on cellular integrity compared to larger PS-MPs (1 µm and 10 µm) [138]. This study, although unique within the reproductive context, is fully consistent with what is widely described in the literature concerning other target organs [165,166], thus emphasizing the importance of also considering nanoparticle size when assessing potential risks to placental health and function [138] (Figure 3).

The impact of PS-MNP deposition or accumulation on fetal health remains poorly understood. Indeed, few studies have investigated the effects of these particles on offspring. It is certain that PS-MNP particles can cross the placental barrier and deposit in fetal tissues following maternal exposure [137,147,152,167].

An ex vivo study has provided compelling evidence that particle size significantly influences their transfer across the placenta. Smaller PS-NPs (50 nm) showed enhanced movement from fetal to maternal circulation, while larger PS-NPs (300 nm) primarily transferred from maternal to fetal circulation. Microscopic analyses confirmed the presence of smaller particles in maternal circulation after fetal perfusion, with larger particles being less detectable [149] (Figure 3). These findings underscore the critical role of particle dimensions in placental transfer dynamics, particularly in understanding the potential fetal exposure to nanoparticles. This knowledge is essential for evaluating the risks and benefits associated with nanoparticle exposure during pregnancy and emphasizes the importance of considering particle characteristics in prenatal toxicology and risk assessment.

#### 3.4.2. Offspring Effects Derived from PS-MNP Maternal Exposure

The effects of exposure to PS-MNPs on offspring have garnered increasing interest in scientific research. Recent studies have highlighted that exposure to PS-MNPs can impact body weight and the proper metabolic development of neonates [140,151,155,156]. Additionally, potential negative effects on lactation have been identified, with implications for the health and development of the offspring. These findings raise significant concerns regarding the long-term effects of PS-MNP exposure on the health and well-being of young organisms.

##### Maternal Exposure to PS-MPs Causes Decreased Offspring Weight

Recent studies suggest a downward trend in average body weights of offspring, potentially linked to environmental factors such as exposure to PS-MNPs. This association was demonstrated in studies where female mouse models were exposed to PS-MNPs during pregnancy [140,147,151].

Specifically, a decreased body weight has been observed in mice born from mothers exposed to PS-MPs throughout the entire gestational period [150,168]. The magnitude of this weight reduction appears to be dependent on both the concentration of PS-MNPs and the sex of the offspring. For instance, exposure to higher concentrations of PS-MNPs resulted in more pronounced decreases in body weight in female offspring compared to male offspring [141].

Furthermore, beyond changes in body weight, exposure to PS-NPs [155] and PS-MPs [168] affected organ weights, particularly impacting liver and testis weights [155,168].

In contrast, some studies have reported no significant effects at low doses, whereas marked disruptions in fetal development were observed in regions heavily polluted with plastics [140]. This emphasizes the critical role of both PS-MNP concentration and the sex of the offspring in determining adverse effects (Figure 3).

##### Maternal Exposure to PS-MNPs Causes Fatty Acid Metabolic Disorders in Offspring

Maternal exposure to PS-MNPs has been shown to induce metabolic disorders, disruption in hepatic transcription, alterations in serum metabolites, and an increased potential risk of metabolic disease in both F1 and F2 offspring. Specifically, gestational exposure to PS-NPs induced non-alcoholic fatty liver disease (NAFLD) in adult female offspring, with no observed effect in males. High doses of gestational PS-NP exposure resulted in hepatic steatosis in adult females compared to males, characterized by increased expression of genes involved in fatty acid uptake and triglyceride synthesis. Additionally, high exposure increased intracellular vacuoles specifically in female offspring livers, without affecting males. Serum alanine aminotransferase (ALT) levels were significantly elevated in high-dose female offspring, suggesting greater susceptibility to adverse effects [140,156]. Detrimental effects were not determined only by the concentration of PS-MNP exposure but were also influenced by their size, highlighting a significant size-dependent relationship. For instance, various studies have indicated that when comparing plastics at the nano and micro scales, nano-scale plastics exhibit higher uptake rates and stronger functional effects. For example, a study showed that with exposure to 5 µm PS-MPs compared to smaller 500 nm particles, male progeny exhibited significant variations in hepatic cholesterol and serum triglyceride levels. Conversely, most serum amino acids decreased in female offspring, while levels tended to increase in male offspring. Moreover, alterations were observed in acyl-carnitine and free carnitine levels, which are important markers for the clinical screening of genetic diseases [151,155,156]. These sex-dependent variations have also been observed by other researchers [140,141,153]. Overall, these changes suggest potential disruptions in fatty acid metabolism and underscore sex-specific reactions to maternal PS-MP exposure during fetal development. Furthermore, the impact of PS-MP exposure was notably reduced in F2 offspring compared to F1 offspring, with effects in F2 generations being considerably milder, characterized by only a few genes showing significant alterations [153] (Figure 3).

The observed metabolic disorders in offspring following maternal exposure to PS-MNPs could be attributed to two potential mechanisms. Firstly, maternal exposure to PS-MNPs may directly alter maternal metabolism, thereby inducing intergenerational effects on the offspring. Secondly, the ability of small-sized PS-MNPs to traverse the placenta and potentially transfer to subsequent generations could contribute to the observed effects. These findings underscore the complex nature of maternal PS-MNP exposure and its impact on metabolic health across generations, highlighting the need for further research to elucidate underlying mechanisms and inform strategies for mitigating potential adverse outcomes.

### 3.5. Post Natal PS-MNP Influence: Effects on Offspring During Lactation

The implications of persistent PS-MNPs during lactation and their subsequent impact on offspring development remain largely unexplored. However, the existing literature evidence suggests that these PS-MNPs have the potential to transfer into breast milk and then orally into offspring, influencing progeny development [145,153,154,155]. This is supported by Huang et al., who demonstrated altered liver morphology and decreased offspring weight over a 3-week lactation period, along with a potential for increased risk of NAFLD [153,155]. Additionally, an increase in the hepatic activities of SOD and CAT was observed, indicating an overproduction of ROS [155]. Comparable effects were observed in offspring testicles, with changes in sperm number and morphology. Notably, PS-NPs ingested by the mother can reach the offspring’s brain through lactation, bypassing the incompletely formed blood–brain barrier (BBB) [145]. This leads to cognitive deficits and detrimental effects on retinal development and function [145,154] (Figure 3).

Overall, these findings highlight the potential risks associated with PS-MNP exposure during lactation, impacting not only systemic physiology but also neurological and developmental outcomes in offspring.

### 3.6. PS-MNPs on Adult Reproductive Health

PS-MNPs have emerged as significant disruptors of mammalian reproductive systems, influencing ovarian and testicular function [17,18,19,20,21,30,31,32,33,34,35]. The paragraphs described subsequently will explore the documented effects of PS-MNPs on female and male reproductive health, highlighting the critical need for further investigation into their long-term impacts.

#### 3.6.1. Effects of PS-MNP Exposure on the Female Mammalian Reproductive System

Exposure to PS-MNPs has shown significant effects on female mammals, influencing various aspects of ovarian and reproductive physiology [17,18,19,30,31,32,33,34]. Key aspects include alterations in ovarian size [31,32,33], reduction of follicular reserve [17,18,19,30,31,32,34] with promotion of advanced stages of apoptosis [18,19,30,32,34], and follicular atresia [34]. Furthermore, significant influence is observed on oocyte meiotic resumption and disruption of hormonal secretion performance [18,19,30,31,32,33]. These factors play a critical role in modulating estrous cycle dynamics [27]. A detailed analysis of these effects is essential to fully understand the impact of PS-MNPs on the female reproductive system.

One of the most notable effects, according to the literature, is that a general decrease in body weight is often observed in exposed subjects [31], which correlates with a reduction in ovarian size and weight [31,33]. Surprisingly, on the contrary, one study indicated that this exposure did not affect ovarian weight and the ovarian index [32], a discrepancy that warrants further investigation to be fully understood.

Exposure to PS-NPs also leads to the development of ovarian fibrosis, marked by an upregulation of fibrosis markers such as fibronectin and collagen I and III [30]. This fibrosis impacts the ovarian stroma and is associated with increased apoptosis in ovarian cells [19,34]. These structural changes can have far-reaching effects on ovarian functionality and overall reproductive health.

A critical aspect of PS-MNP exposure is its detrimental effect on the follicular reserve. Studies have consistently shown a significant reduction in the number of growing follicles at all stages [17,18,19,31,32,34]. This depletion is closely linked to increased apoptosis and atresia within ovarian follicles. The heightened rate of apoptosis damages granulosa cells, reducing their thickness and compactness [18,34]. Consequently, this impairs follicular development and leads to increased atresia, as evidenced by the observed corpus luteum atrophy and rise in atretic follicles [19,31,34]. This chain of events results in a compromised ovarian reserve and diminished ovarian functionality.

The negative impact of PS-NPs extends to the germinal compartment, where they disrupt the acquisition of meiotic competence. This is evident in reduced oocyte survival rates and compromised transitions from the germinal vesicle breakdown (GVBD) phase to metaphase II (MII) during oogenesis [17]. The interruption of these critical processes further undermines reproductive potential.

The interference of hormonal balance is another significant consequence of PS-MNP exposure. Most studies report a downregulation of luteinizing hormone (LH), anti-Müllerian hormone (AMH), and progesterone (P4), coupled with an increase in testosterone, estradiol (E2), and follicle-stimulating hormone (FSH) levels [18,19,30,31,32]. These hormonal disruptions are complex, with some studies presenting conflicting results [33,146], suggesting intricate interactions that need more exploration.

**Figure 4:**
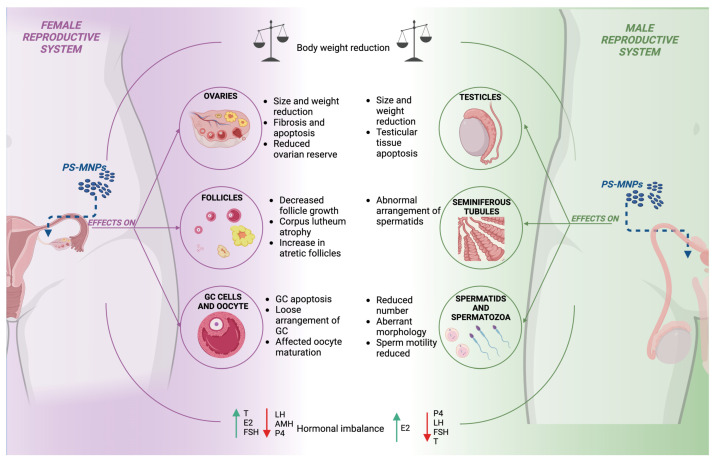
Effects of PS-MNP exposure on male and female reproductive systems. Image created with Biorender.com (accessed on 26 July 2024).

These physiological and hormonal disturbances inevitably impact estrous cycle dynamics. Changes in body and ovarian weight, follicular depletion, increased apoptosis, and hormonal imbalances collectively shorten the estrous cycle duration [19]. This shortening can be associated with difficulties in embryo implantation [19,31], reduced fertility, and increased spontaneous fetal losses [32] (Figure 4). Understanding these dynamics is crucial for assessing the broader implications on reproductive health.

In summary, the exposure to PS-MNPs results in a cascade of effects starting from structural changes in the ovaries to significant disruptions in follicular health, meiotic processes, and hormonal balance. These interconnected effects culminate in altered estrous cycles and compromised reproductive performance. These findings underscore the importance of continued research to fully understand the long-term impacts of PS-MNPs on female reproductive health.

#### 3.6.2. Effects of PS-MNP Exposure on the Male Mammalian Reproductive System

Exposure to PS-MNPs has been shown to interfere with the normal process of spermatogenesis in the testicles, compromising the production, quality, and maturation of spermatozoa. This disruption may have significant implications for male fertility and reproductive health [20,21,31,35].

Interestingly, it has been observed that the absorption of PS-MPs is lower in the testicles compared to the ovaries [31]. Specifically, the accumulation in females is approximately 30% higher than in the testicles. This suggests a significant difference in the biodistribution and retention of PS-MPs between male and female gonadal tissues. Further studies are needed to understand the underlying mechanisms driving this disparity, which could have implications for gender-specific therapeutic applications and safety assessments of PS-MPs [31].

Similarly to what was observed in females, a decrease in overall body weight [20,21,31] and a reduction in reproductive organ weight have been noted in males as well [21,31,35], indicating a systemic impact of PS-MNP exposure.

Histological analysis has revealed that exposure to PS-NPs [35] and PS-MPs [20] induces apoptosis in testicular tissues [35], leading to the formation of empty cavities that become more pronounced at higher doses, potentially impairing sperm production and fertility [20,35]. In fact, the abnormal organization of spermatids within the seminiferous tubules has been noted, characterized by a disordered and loosely compacted arrangement [21,31]. These spermatids, in reduced quantities [31,35], also exhibited compromised morphology, marked by signs of pyknosis and nuclear fragmentation [35]. Furthermore, studies have revealed a significant decrease in the number of viable sperm in the epididymis following exposure to PS-NPs [35] and PS-MPs [20,31], accompanied by reduced motility and an increase in morphological abnormalities such as acrosome absence, cervical folding, acephaly, and small head, or tailless [20,21,31,35]. These anomalies were directly correlated with damage to sperm precursors.

It also has been highlighted that PS-MPs affect energy metabolism and sperm development, resulting in a decrease in the activity of the sperm metabolism-related enzymes, succinate dehydrogenase and lactate dehydrogenase [20]. Supporting evidence from another study suggests that PS-MPs may compromise the blood–testis barrier (BTB); apparently, this explains why the number of sperm abnormalities increased and the quality of sperm decreased [21].

From an endocrine perspective, hormonal changes have been observed that may significantly impact male reproductive function. Specifically, exposure to PS-MPs has affected male hormone levels, resulting in a decrease in progesterone, LH, FSH, and testosterone, and an increase in E2 compared to non-exposed subjects [20,21,31] (Figure 4).

Although the effects were less evident compared to female subjects, where there was a greater impact on fertility and embryonic development, the effects on the male reproductive system remain significant. In conclusion, exposure to PS-MNPs compromises male reproductive functions by affecting sperm production and quality and disrupting the hormonal balance essential for fertility.

### 3.7. Mechanism of Action of PS-MNPs in Reproduction Systems

Understanding the role of oxidative stress and inflammation signaling pathways in PS-MNP-induced reproductive function failures is crucial. Oxidative stress, characterized by an imbalance between oxidation and antioxidant defenses, emerges as a key factor in the toxicity of these nanoparticles, significantly affecting fertility [169,170]. PS-MNPs modulate the expression of genes and proteins critical for antioxidant and inflammatory responses, leading to cellular and molecular changes that impair reproductive function [35,171,172,173]. Grasping these mechanisms is essential for fully assessing the impact of PS-MNPs on reproductive health and for developing effective mitigation strategies.

#### 3.7.1. The Role of Signaling Pathways of Oxidative Stress and Inflammation in PS-MNP-Mediated Male and Female Reproductive Function Failures

Oxidative stress, characterized by an imbalance between the body’s oxidation and antioxidation defense mechanisms, emerges as a critical factor in the pathophysiology of infertility, affecting both male and female reproductive systems. The literature demonstrates that the induction of oxidative stress represents the primary mechanism of toxicity associated with PS-MNPs, both in vivo and in vitro [174,175].

The response to stress stimuli, such as oxidative stress, is mediated by groups of molecules that participate in intracellular signaling pathways [176,177]. These molecules convey the message from the external environment to the interior of the cell, and their activation is regulated at the transcriptional level [176,177]. Several genes have been described as responsible for the fine-tuning of the oxidative stress response to exposure to PS-MNPs [35,171,172,173]. Among these are nuclear factor erythroid 2–related factor 2 (Nrf2), Heme oxygenase-1 (HO-1), Superoxide dismutase (SOD), Glutathione peroxidase (GPx), Catalase (CAT), Glutathione (GSH), and NADPH oxidase 4 (NOX4) [176,177]. Each of these genes has a specific function in regulating ROS. These genes and their encoded proteins play integral roles in the cellular signaling pathways that enable responses to oxidative stress, thereby protecting cells from the damage induced by free radicals and other reactive oxygen species. It has been observed that PS-MNPs induce oxidative stress in granulosa cells, leading to the upregulation of NOX4, a gene involved in ROS generation, while GPx, responsible for ROS elimination, is strongly downregulated [32]. Furthermore, it has been observed that ROS induced by PS-NPs [32] and PS-MPs [34] activate the Hippo pathway, a pathway normally related with physiological and pathological ovarian aging, through the MST1-LATS1 signaling cascade, leading to downstream signaling changes and cellular apoptosis, resulting in decreased fertility [32,34].

This oxidative stress, in addition to directly influencing granulosa cell function, has systematic effects on the entire ovarian antioxidant system, as evidenced by increased levels of malondialdehyde (MDA) [18,19]. However, several studies have also reported an increase in CAT and SOD levels, indicating an attempt by the antioxidant defense system to counteract excessive ROS production [17]. The increase in CAT and SOD may not be sufficient to fully compensate for oxidative stress, as suggested by decreased levels of reduced GSH, indicating an overall deterioration of the ovarian antioxidant system [17,33,178].

At the molecular level, several pathways have been described as active and modulated in response to oxidative stress. NF-κB is a key transcription factor in regulating the cellular inflammatory response, controlling the secretion of inflammatory cytokines such as IL-6, IL-1β, and TNFα [179,180]. Similarly, Nrf2 is crucial for the antioxidant defense of cells, regulating signaling pathways such as HO-1 to protect against oxidative stress [173,181]. Recently, it has been discovered that Nrf2 also plays a significant role in the inflammatory response [172]. Activation of Nrf2 can increase the expression of HO-1, which inhibits NF-κB and reduces inflammation [171]. However, NF-κB can inhibit Nrf2 signaling, creating a reciprocal regulatory dynamic. Recent studies have demonstrated that exposure to PS-MNPs induces inflammation by activating NF-κB and increasing inflammatory cytokines while decreasing levels of anti-inflammatory factors such as Nrf2 and HO-1 [35].

Furthermore, studies have shown that when cells undergo oxidative stress, they can induce and activate the release of the NLRP3 inflammasome, an inflammation-associated factor, by activating Caspase-1, leading to the maturation of proinflammatory cytokines [182,183,184]. Specifically, the activation of Caspase-1 induces pyroptosis [185]. Following pyroptosis activation, there is an overproduction of proinflammatory cytokines that cause tissue damage. It has been observed that PS-NPs [17,18] and PS-MPs [20,21] can activate these pathways and significantly increase levels of cytokines such as IL-1β, IL-6, IL-18, and other inflammatory factors such as TNFα, MCP1, and CXCL10, leading to an inflammatory state in target compartments such as ovaries [17,18] and testicles [20,21].

Another important aspect that has been observed is that oxidative stress has been shown to activate the Wnt/B-catenin signaling pathway in the ovary and granulosa cells, promoting the expression of genes related to fibrosis [30]. This process can lead to the stimulation and proliferation of fibroblasts, resulting in differentiation into myofibroblasts capable of synthesizing high amounts of collagen I and III, thereby contributing to ovarian fibrosis [30].

**Figure 5:**
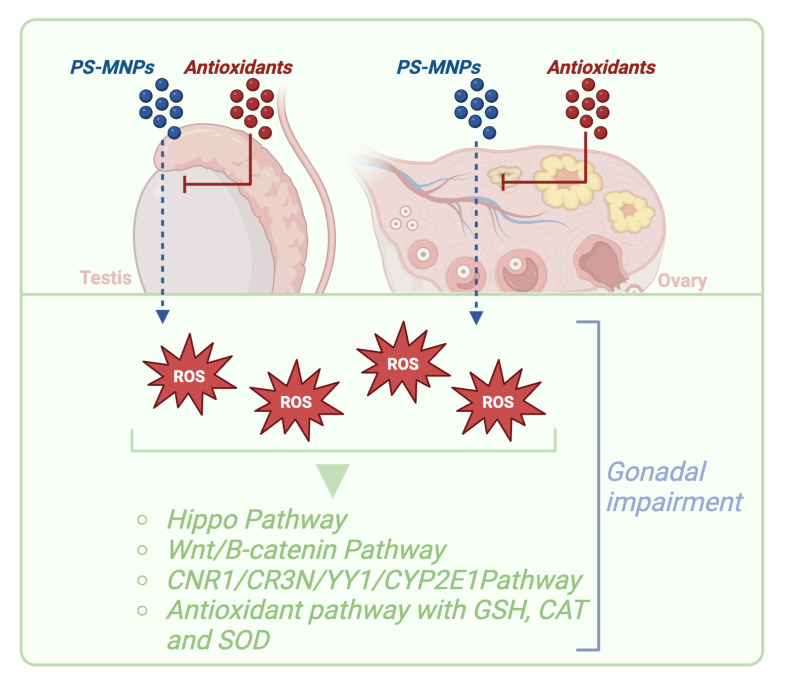
Impact of PS-MNP exposure on gonadal oxidative stress pathways: intracellular pathways activated in response to oxidative stress induction in the gonads by PS-MNP exposure. Exposure to PS-MNPs promotes the increase in reactive oxygen species (ROS), initiating a cascade of intracellular signaling that compromises redox homeostasis and gonadal function. Antioxidant intervention inhibits these pathways, mitigating the detrimental effects of oxidative stress on the gonads. Image created with Biorender.com (accessed on 26 July 2024).

Additionally, another pathway activated by oxidative stress is the CNR1/CR3N/YY1/CYP2E1 path, which can cause oxidative damage to ovarian DNA [34] (Figure 5).

In summary, the accumulation of ROS and alteration of antioxidant enzymes in the ovarian context are significant indicators of oxidative stress caused by exposure to PS-MNPs, with relevant consequences for ovarian health and fertility. This phenomenon is not limited to the female gender alone; indeed, studies conducted on males have shown elevated levels of ROS and MDA, along with a decrease in GSH after exposure to PS-MPs, consistent with findings in studies conducted on females [20,31]. This indicates that the effect of PS-MPs on oxidative stress is relevant for both male and female reproductive systems, with significant implications for health and fertility in both sexes.

#### 3.7.2. Are Antioxidants the Key to Prevent PS-MNP Effects on Reproductive Systems?

Based on these premises, the use of antioxidants emerges as a potential therapeutic strategy to counteract the damage caused by PS-NPs [30,32] and PS-MPs [20] to the reproductive system. Antioxidants, by acting as scavengers of free radicals, can neutralize excess ROS generated by exposure to both PS-NPs [30,32] and PS-MPs [20], thus protecting ovarian and testicular cells from oxidation and reducing the risk of infertility [20,30,32]. Additionally, antioxidants can contribute to restoring compromised cellular redox balance, supporting reproductive function and the quality of sperm and oocytes. Therefore, the targeted use of antioxidants may represent an effective strategy for preserving reproductive health and promoting fertility in individuals exposed to PS-MNPs [20,30,32].

Among the most promising antioxidant agents tested to date with potential applications for reversing the detrimental effects of PS-NP exposure, we find salidroside, a natural antioxidant, that has been shown to alleviate the adverse effects of PS-NPs on granulosa cells by drastically reducing ROS accumulation [32]. Therefore, this compound has therapeutic potential for preventing and/or reversing ovarian damage induced by PS-NPs [32].

The administration of N-Acetyl-L-cysteine (NAC) as an antioxidant has also significantly attenuated the increase in ROS as shown in studies conducted on both male [20] and female subjects [30] (Figure 5).

Exposure to PS-MNPs can have deleterious effects on both male and female reproductive systems. These studies have highlighted that these PS-MNPs can cause an increase in oxidative stress and inflammation in reproductive tissues, compromising the function of the involved organs. In the case of the male reproductive system, this results in decreased sperm quality and fertility, while in the female reproductive system, it negatively affects oocyte maturation and conceiving capability. These effects can have significant consequences on reproductive health and fertility. Therefore, it is essential to deepen the understanding of the mechanisms through which PS-MNPs influence the reproductive system to develop targeted preventive and therapeutic strategies to protect both male and female reproductive health.

##### Beneficial Reproductive Effects of Antioxidants Derived from Biological Matrices

**Figure 6:**
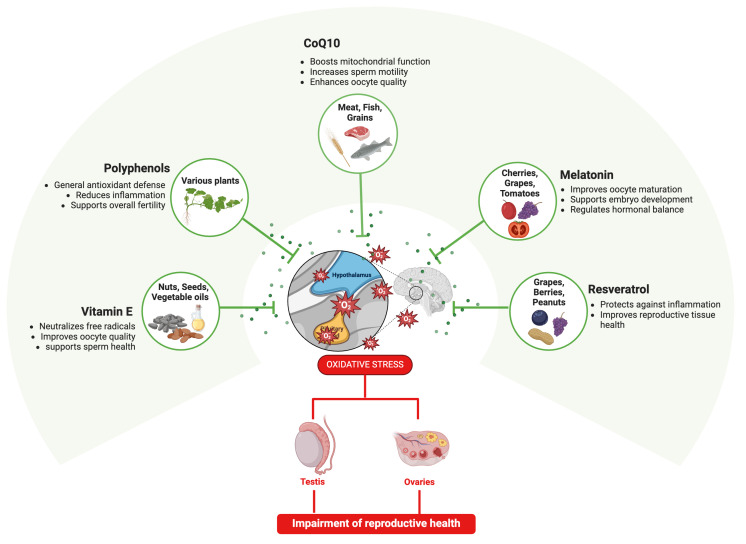
Potential use of biological matrix-derived antioxidants to combat oxidative stress from microplastic exposure: implications for ovarian health and reproductive function. Image created with Biorender.com (accessed on 26 July 2024).

The potential use of exogenous matrices as antioxidants to protect or rescue fertility is an emerging area of interest. The beneficial effects have been studied on reproductive conditions caused by PS-MNPs [186,187], recognizing an oxidative stress-mediated mechanism on reproductive outcomes in both male and female fertility [27,29,188,189]. In this context, promising evidence of the efficacy of antioxidant-rich biological matrices—derived from natural foods, supplements, or specialized diets—in mitigating oxidative stress have been collected to date [190], starting to detail the mechanisms by which dietary-induced oxidative stress exacerbates endocrine disruption. Specifically, antioxidant-rich biological matrices may be enriched in vitamin E [191], coenzyme Q10 (CoQ10) [191], melatonin [192], and polyphenols like resveratrol [193]. These are bioactive components belonging to natural sources such as fruit, vegetables, nuts, and grains. More in detail, Vitamin E, present in nuts, seeds, and vegetable oils, has been shown to protect sperm and oocytes from oxidative damage, and acts as a potent antioxidant that neutralizes free radicals and reduces oxidative stress in reproductive tissues [191,194]. Coenzyme Q10, which is abundant in meat, fish, and whole grains, improves mitochondrial function and has been associated with enhanced sperm motility and oocyte quality [191]. Melatonin, a hormone with antioxidant properties, is naturally found in small amounts in foods like cherries, grapes, and tomatoes and has been reported to improve oocyte maturation and embryo development [192]. Polyphenols such as resveratrol, present in grapes, berries, and peanuts, have demonstrated protective effects against oxidative stress and inflammation in reproductive tissues [193] (Figure 6). Drawing from these findings, it is plausible that the same antioxidants could mitigate the reproductive toxicity caused by PS-MNP exposure, even if targeted investigations are needed to discover the impact of different antioxidant natural compounds on PS-MNP-induced oxidative stress on reproductive cells/tissues, defining the mechanisms related to antioxidant dosages, delivery methods (i.e., their interactions with other dietary components), and long-term effect benefits.

## 4. Discussion

In this review, we elucidate the significant adverse effects of PS-MNPs on mammalian reproductive health, which stem primarily from oxidative stress, inflammation, and endocrine disruption. These findings indicate that PS-MNP exposure can disrupt endocrine control, affecting reproductive systems in both males and females. Beyond the systemic effects that could potentially be mitigated by hormone therapy, PS-MNPs impact gonads directly, influencing both somatic and germ cell compartments. For instance, prolonged exposure to PS-MNPs in females is associated with premature ovarian aging, driven largely by apoptosis, while in males, similar exposure impairs spermatogenesis, reducing sperm quality and even leading to azoospermia.

Recent studies have enriched our understanding of how apoptosis, inflammation, and oxidative stress interact to affect reproductive health, potentially offering insights for managing infertility linked to PS-MNP exposure. Apoptosis, a significant gonadal response to toxic stimuli like PS-MNPs, is particularly concerning in cases of prolonged exposure, as it can drive reproductive failure, especially when compounded by chronic inflammation. Inflammation, which plays critical roles in reproductive processes such as ovulation, implantation, and spermatogenesis, is thus a likely contributor to infertility.

The oxidative stress that exacerbates inflammation results from an imbalance between reactive oxygen species (ROS) generated by PS-MNPs and antioxidant defenses in reproductive cells. This interplay between oxidative stress, inflammation, and apoptosis forms a critical pathway underlying PS-MNP-mediated infertility, which future studies could address by isolating the effects of physical PS-MNP particles from those of the endocrine-disrupting chemicals they release upon degradation.

Research gaps remain in understanding the specific impacts of PS-MNPs, particularly regarding dose thresholds, chronic impacts, and the potential for intergenerational transmission of PS-MNP effects. There is also a need for advanced methodologies, such as long-term in vivo studies and in vitro cellular models, that can isolate the impacts of PS-MNPs on different reproductive tissues. Understanding these nuanced effects, as well as the unique vulnerabilities of male and female reproductive systems, will be essential in developing comprehensive risk assessments and protective guidelines for exposure to environmental MNPs.

## 5. Conclusions

Our findings, together with previous evidence, emphasize that PS-MNPs pose a substantial risk to reproductive health, primarily through pathways of oxidative stress, inflammation, and endocrine disruption. The evidence suggests that the physical presence of PS-MNPs, as well as the toxic chemicals released during their degradation, significantly impact reproductive health, with consequences that span across generations. These risks underscore the importance of investigating both the immediate effects of PS-MNPs and the role of chemical leachates in reproductive toxicity. Given the increasing prevalence of PS-MNPs in the environment, addressing these research gaps will be vital to developing interventions and regulatory frameworks aimed at mitigating the reproductive risks associated with PS-MNP exposure. These insights can inform public health policies and support the development of more stringent environmental regulations to manage the impact of MNPs on reproductive health.

## Figures and Tables

**Table 1:** A comprehensive overview of experimental conditions for assessing nanoplastic exposure: facilitating comparison and understanding across different approaches. This table provides a detailed summary of studies involving PS-MNP exposure, indicating first whether the study was conducted in vivo or in vitro. For in vivo and in vitro studies, the animal model used is specified, while for in vitro studies, the type of sample is described. Following this, the table lists the size of the PS-MNPs, the dose administered, the exposure time, and the mode of exposure in vivo.

In Vivo/In Vitro Studies	Species	Sample Typology for In Vitro Investigation	PS-MNP Size	PS-MNP Dose	In Vivo Exposure Time	In Vivo Exposure Way	References
In vitro/in vivo	Mouse	Human granulosa cell line KGN	20 nm	100 μg/mL in vitro 1 mg/d in vivo	48 h in vivo	Oral gavage	[32]
In vivo	Rat	/	800 nm	From 2.5 to 10 mg/kg/day	45 days	Oral administration	[33]
In vivo	Mouse	/	50 nm and 5 μm	100 ng/L	All gestation (20 days)	Drinking water	[139]
In vivo	Mouse	/	80 nm	From 1 to 25 μg/µL	All gestation (20 days)	Inhalation	[140]
In vivo	Mouse	/	80 nm	From 1 to 25 μg/µL	All gestation (20 days), 3 times per week	Oropharyngeal aspiration	[141]
In vivo	Mouse	/	50 nm and 500 nm	From 0.5 to 1000 µg/day	From embryonic day 8 (E8) until 2 weeks after birth	Oral administration	[145]
In vitro	Human	BeWo b30 choriocarcinoma cell line (placental cells)	200 nm, 500 nm, 1 µM, and 10 µM	100 µg/mL	/	/	[138]
In vitro	Swine	Granulosa cells	100 nm	From 5 to 75 µg/mL	/	/	[146]
In vivo	Rat	/	20 nm	300 μL (2.64 × 10^14^ particles)	On gestational day 19 for 24 h	Intratracheal instillation	[147]
In vivo	Mouse	/	5 µm	From 100 µg/L to 10 mg/L	35 days	Drinking water	[35]
In vivo	Rat	Granulosa cells	500 nm	From 0.015 to 1.5 mg/kg/day	90 days	Drinking water	[18]
In vivo	Mouse	/	5 μm	From 0.01 to 1 mg/day	42 days	Oral gavage	[20]
In vivo	Mouse	/	10 μm	250 μg in a 200 μL saline solution	On days 5.5 and 7.5 of gestation	Intraperitoneally injected	[148]
In vivo/in vitro	Rat	Granulosa cells	500 nm	From 0.015 to 1.5 mg/day In vivo From 1 to 25 μg/mL in vitro	90 days (in vivo); 24 h (in vitro)	Drinking water	[30]
In vivo	Mouse	/	5 μm	0.1 mg/day	20 days	Oral gavage	[31]
In vivo	Mouse	/	700 nm	30 mg/kg	35 days	Oral gavage	[17]
In vivo	Mouse	Granulosa cells	5 μm and 10 µm	100 mg/L	35 days	Drinking water	[34]
In vivo/in vitro	Mouse	Germ cells (GC), Leydig cells (LC), and Sertoli cells (SC)	500 nm, 4 μm, and 10 μm	10 mg/mL	28 days (in vivo); 24 h (in vitro)	Oral gavage	[21]
In vitro	Human	BeWo b30 choriocarcinoma cell line (placental cells)	50 nm and 300 nm	25 μg/mL	Perfusion for 6 h	/	[149]
In vivo	Mouse	/	10 μm and 150 μm	From 0.4 to 40 mg/kg/day	30 days	Oral gavage	[150]
In vitro/in vivo	Mouse	Mouse ovarian tissue and human ovarian granulosa cell lines	50 nm	From 5 mg/Kg to 25 mg/Kg in vivo; from 50 to 200 μg/mL in vitro	8 weeks	Oral administration	[19]
In vivo	Mouse	/	500 nm and 5 µm	100 µg/L and 1000 µg/L	All gestation (20 days)	Drinking water	[151]
In vivo	Mouse	/	50 nm	From 50 to 200 µg/mL	35 days	Oral gavage	[152]
In vivo	Mouse	/	5 µm	100 and 1000 µg/L	During pregnancy and lactation (6 weeks)	Drinking water	[153]
In vivo	Mouse	/	100 nm	2.07 × 10^10^ particles mL^−1^	From the first day of pregnancy until the end of lactation (21 days after birth)	Drinking water	[154]
In vitro/in vivo	Mouse	Trophoblast cells	From 20 nm to 500 nm	300 μg	4 h	Injection through jugular vein	[137]
In vivo	Mouse	/	100 nm	From 0.1 mg/mL to 10 mg/mL	21 days	Drinking water	[155]
In vivo	Mouse	/	80 nm	0.5 µg/µL and 1 µg/µL	21 days	Inhalation	[156]
